# Matched pair analysis of wide versus narrow focus during shockwave lithotripsy for urolithiasis

**DOI:** 10.1007/s00240-024-01682-0

**Published:** 2024-12-21

**Authors:** Anna J. Sharp, Catherine E. Lovegrove, Roshan Sreekumar, Mandy Spencer, Benjamin W. Turney, Sarah A. Howles

**Affiliations:** 1 Department of Urology, Oxford University Hospitals NHS Trust, Oxford, Oxfordshire UK; 2 Imperial College Healthcare NHS Trust, London, UK; 3 Nuffield Department of Surgical Sciences, University of Oxford, Oxfordshire, OX3 9DU UK; 4 Department of Radiology, Oxford University Hospitals NHS Trust, Oxford, Oxfordshire UK

**Keywords:** Nephrolithiasis, Shockwave lithotripsy, Focus size, Stone clearance, Complications

## Abstract

**Purpose:**

To compare stone clearance and complications between a ‘wide’ (9 × 50 mm) and ‘narrow’ shockwave focus (6 × 28 mm) when undertaking shockwave lithotripsy (SWL) in patients with renal or ureteric stones.

**Methods:**

Data from patients undergoing SWL using the dual focus Storz Modulith SLX-F2 lithotripter at a single centre were prospectively collected between February 2018 and September 2020. Patients were matched by stone size, location, and number of treatments. Stone clearance, re-presentation within 31 days, symptoms, complications, and need for post SWL-interventions were compared using McNemar’s test.

**Results:**

Patients receiving wide focus SWL (WF-SWL, *n* = 152) were matched with patients receiving narrow focus SWL (NF-SWL, *n* = 152). Median stone size was 6 mm; energy delivered to WF-SWL and NF-SWL groups was comparable. Complete stone clearance was achieved in 55% of WF-SWL patients (*n* = 84) and 41% (*n* = 63) of NF-SWL patients (*p* = 0.04). Treatment was considered successful in 74% (*n* = 113) of WF-SWL cases and 66% (*n* = 100) of NF-SWL (*p* = 0.20). No difference in rates of readmission, post-procedural pain, haematuria, urinary tract infections, analgesia or antibiotic requirements were identified.

**Conclusion:**

This service evaluation demonstrates no differences in rates of overall treatment success nor complications on comparing WF-SWL and NF-SWL.

## Introduction

The prevalence and incidence of kidney stone disease is rising [1–3] and shockwave lithotripsy (SWL) offers a non-invasive alternative to surgical treatment strategies for ureteric and renal calculi. SWL aims to fragment stones by delivering focal repetitive shockwaves, inducing shear stress, spallation and cavitation of the calculi [4–6]. Although SWL is accepted as a safe and well-established treatment, complications associated with this treatment modality include pain, haematoma, infection, and ureteric obstruction by stone fragments. Despite progressive evolution of lithotripter design, mounting evidence suggests that newer second and third-generation models may offer little benefit over the original first-generation Dornier HM3 lithotripter [7–9] and there is a lack of consensus regarding optimal settings for parameters including the number, frequency, energy level, and focus size of delivered shocks.

Some lithotripters enable a choice of shockwave focus size, whilst several newer generation lithotripters offer only a small focus size [10]. Preclinical studies of efficacy of stone fragmentation with wide- vs. narrow-focus report conflicting results and favourable clinical outcomes from a smaller focus size have not been consistently demonstrated. Due to a lack of evidence, current international guidelines do not give any recommendations regarding the focus size of shockwave delivery [11, 12].

In this service evaluation, we aimed to ascertain whether wide- or narrow-focus SWL offers superior stone clearance and complication profiles using a matched-pair study design.

## Patients and methods

### Data collection

**Fig. 1:**
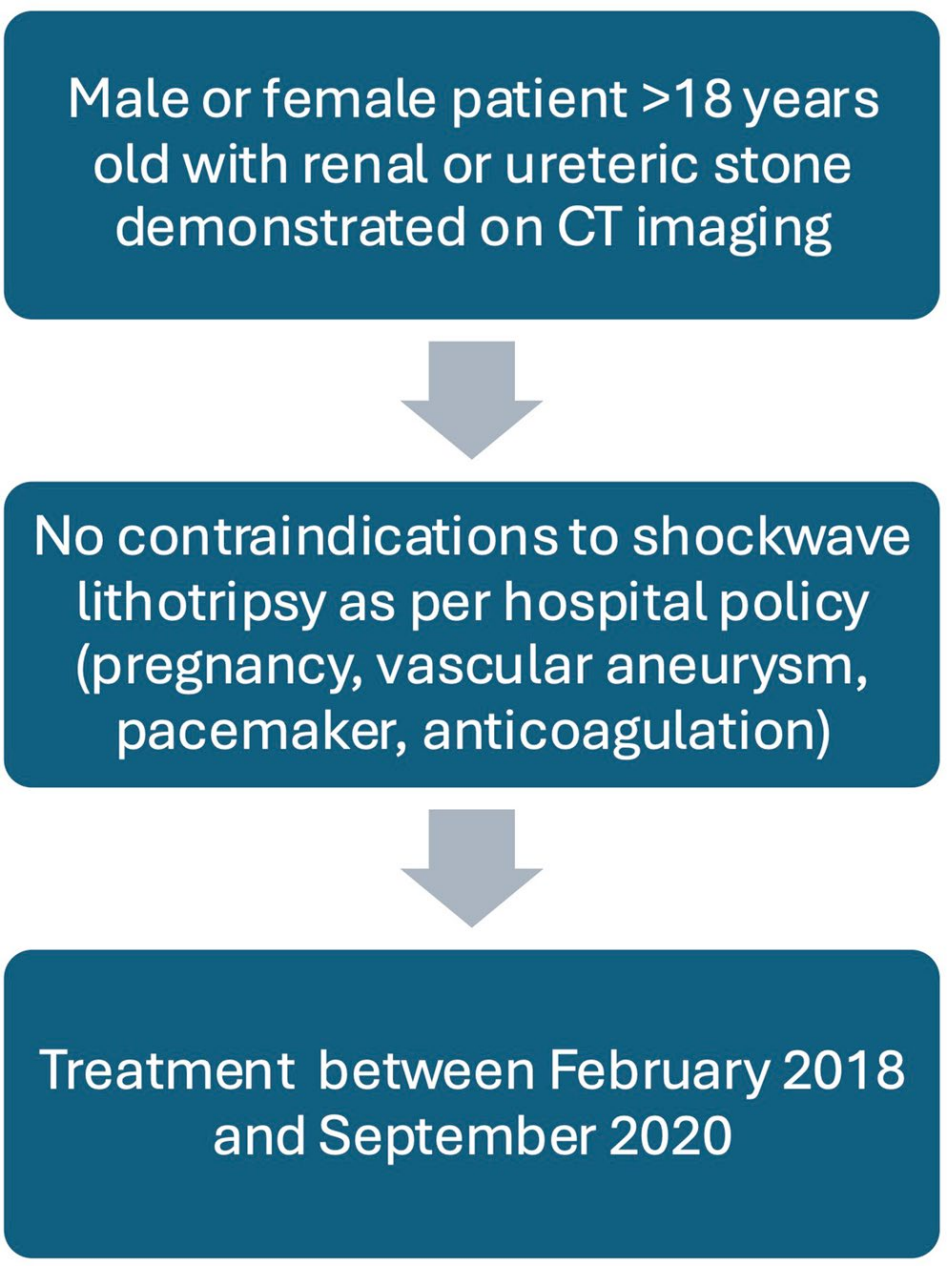
Flow diagram denoting inclusion criteria for analysis

A retrospective analysis of a prospectively collected database was performed (Fig. 1). The database was established to enable internal audit and improve clinical care. Inclusion criteria for this analysis were any patient over 18 years old with a renal or ureteric calculus who underwent SWL at a single, tertiary urology centre between February 2018 and September 2020. Contraindications to treatment included pregnancy, use of anticoagulant therapy, and presence of an intra-abdominal vascular aneurysm or presence of a pacemaker.

All cases were treated with a Storz Modulith SLX-F2 (Storz Medical, Kreuzlingen, Switzerland) third-generation electromagnetic lithotripter. The Storz Modulith SLX-F2 lithotripter allows a choice of two focus sizes: narrow (6 × 28 mm, NF-SWL) and wide (9 × 50 mm, WF-SWL). All patients received 2000 shocks at 1.0 Hz, energy at initiation of therapy was 1.0 (Storz units) and increased to a maximum of 6.0 for renal stones and 8.0 for ureteric stones depending on tolerance. The Storz Medical Lithotripsy Index (SMLI), a measure of total energy delivered based on number of shockwaves and energy per shock, was recorded at the end of each treatment session. Between February 2018 and July 2019, all patients requiring SWL received treatment with the narrow focus setting. From August 2019 to September 2020, a wide focus was used exclusively due to a change in local standards of care, except in cases where patients had already commenced treatment with using a narrow focus in the preceding time period. Imaging was performed between each round of SWL to assess changes to stone burden. This was typically a plain x-ray, unless the stone was radiolucent and ultrasound was used to guide treatment, or where the follow-up x-ray was unclear; in these instances computerised tomography (CT) was used to assess stone position and size. The need for further treatment or an outcome of treatment success was decided on imaging review by a multidisciplinary team comprising urologists, a radiologist, a radiographer, and a nurse specialising in kidney stone treatment (Table 1). Where no residual fragments were visualised, this data was recorded. Data was excluded from analysis where follow-up data was incomplete or where individuals had not reached 2000 shocks during one SWL treatment. If patients had multiple calculi, these were treated on separate occasions. For the purposes of data collection, only the target stone was considered which was the most distal (if ureteric), or closest to the renal pelvis (if renal). Calculi of 3 mm or smaller were not treated.

**Table 1:** Outcome measures used to assess success of clearance and resulting complications, and other relevant definitions

Outcome measure	Definition
STONE CLEARANCE	
*Treatment Outcome*	
Complete stone clearance	Lack of residual fragments on follow-up plain radiograph, ultrasound, or non-contrast CT as reported by a consultant radiologist (chosen imaging modality depended on clinical context)
Treatment success	Stone clearance achieved, or insignificant post-procedural fragments remaining (< 4 mm in size and patient asymptomatic). These patients were not offered further treatment
Persistent stones post SWL	Stones ≥4 mm post treatment (typically after two unsuccessful rounds of SWL). Patients seen in a dedicated stone clinic to discuss further treatment or surveillance
COMPLICATIONS	For inclusion, representation or complications occurred within 31 days of patients’ last round of SWL
*Re-presentation*	
Unplanned healthcare reattendance	Presentation to emergency department or urology triage with symptoms potentially secondary to SWL (such as flank pain, dysuria, haematuria). This also included patients who had had a urine culture specimen sent from their primary care practitioner for a suspected UTI
Readmission required	Patients readmitted to hospital with complications potentially secondary to SWL (urosepsis, haematuria, severe pain, etc.). *NB– such patients were counted both under representation and readmission*
Admission post-SWL	Patients who became acutely unwell either during or shortly after SWL/prior to departure from the unit and were directly admitted
*Reported Symptoms*	
Pain	Patient-reported pain deemed likely attributable to SWL, including flank pain or ‘loin to groin’ pain ipsilateral to SWL site, or dysuria
Fever	Recorded pyrexia of 38.0 degrees centigrade and above
Haematuria	Visible haematuria only
Other	Any other relevant symptoms
*Identified Complications*	
Infection	Symptomatic dysuria with a positive mid-stream urine (MSU) culture or indicative urine dipstick (nitrites and leucocytes) where MSU not available
Haematoma	Intraparenchymal or perinephric, identified on imaging
Other	Any other relevant complications such as obstruction of the urinary tract by remnant fragments, hydronephrosis, etc.
None	Total of the patients who re-presented to hospital in whom no specific complication was identified, in addition to the patients who did not re-present at all

Other terminology	Definition
Kidney stones	All renal stones up to/including the renal pelvis
Ureteric stones	All ureteric stones up to/including the pelvi-ureteric junction (PUJ)
Proximal ureter	Ureter proximal to sacroiliac joint
Distal ureter	Ureter distal to sacroiliac joint

Stone size, laterality, position within the urinary tract, number of lithotripsy treatments administered, efficacy of stone clearance (Table 1), occurrence of complications, requirement for analgesia, antibiotics, and further procedures under anaesthetic following SWL were prospectively recorded.

### Statistical analyses

Patients were matched in a 1:1 ratio for SWL focus size according to the number of SWL treatments, the size of stone treated, and the site of the stone within the kidney or ureter. Wilcoxon signed-rank test with continuity correction was used to assess whether there was a significant difference in energy delivered between matched pairs. McNemar’s test was used to compare measures of effectiveness and complication rates between wide and narrow focus matched pairs. All statistical calculations were undertaken in R (v4.1.1) and p-values reported after correction for multiple testing using a Bonferroni adjustment. A p-value threshold of < 0.05 after correction for multiple testing was used to infer statistical significance.

### Ethical approval

This analysis of data from clinical care was reviewed by the Joint Research Office University of Oxford and the Oxford University Hospitals NHS Foundation Trust and judged to be a description of the findings of clinical service.

## Results

### Demographics

**Fig. 2:**
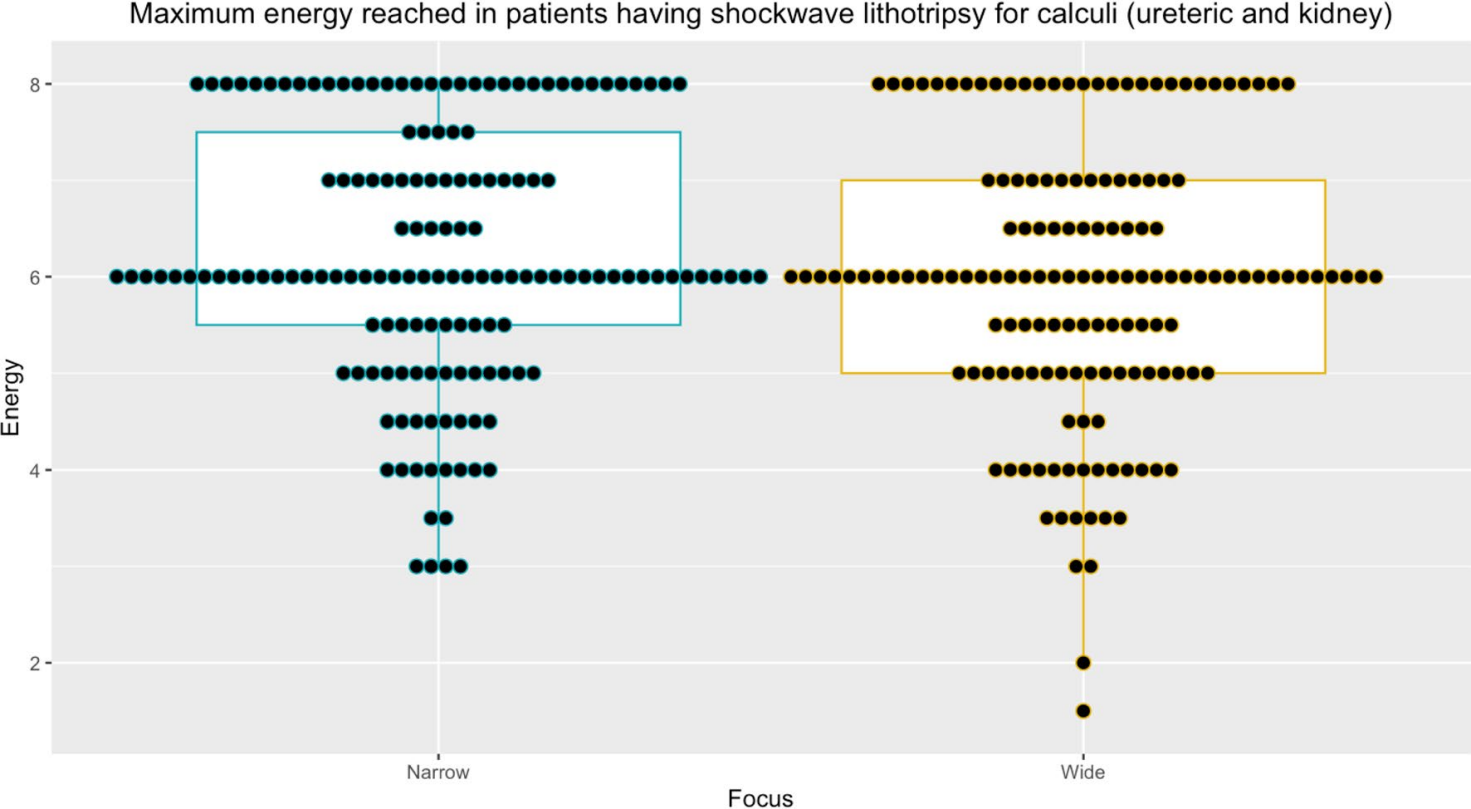
Comparison of maximum energy reached between combined wide and narrow focus SWL treatment groups (*n* = 152 pairs). Median maximum energy reached = 6.00 (IQR 5.00–7.00), median maximum energy reached in narrow-focus subset group = 6.00 (IQR 5.00-7.50), median maximum energy reached in wide-focus subset group = 6.00 (IQR 5.00–7.00). P narrow vs. wide = 0.24

**Fig. 3:**
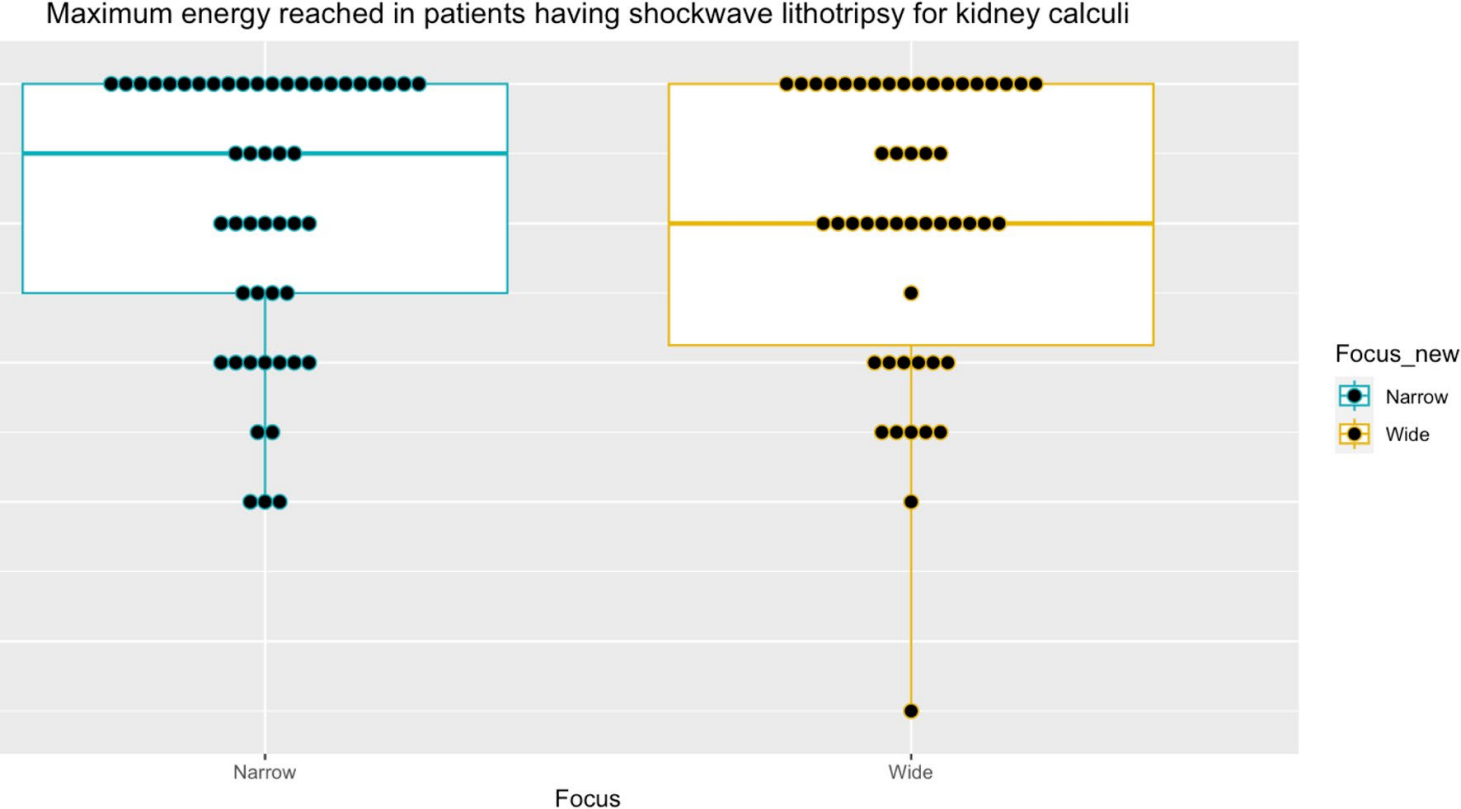
Comparison of maximum energy reached between wide and narrow focus SWL in the kidney subset groups (*n* = 50 pairs). Median maximum energy reached in kidney subset group = 5.25 (IQR 4.38-6.00), median maximum energy reached in narrow-focus kidney subset group = 5.50 (IQR 4.50-6.00), median maximum energy reached in wide-focus kidney subset group = 5.00 (IQR 4.13-6.00). P narrow vs. wide = 1.00

**Fig. 4:**
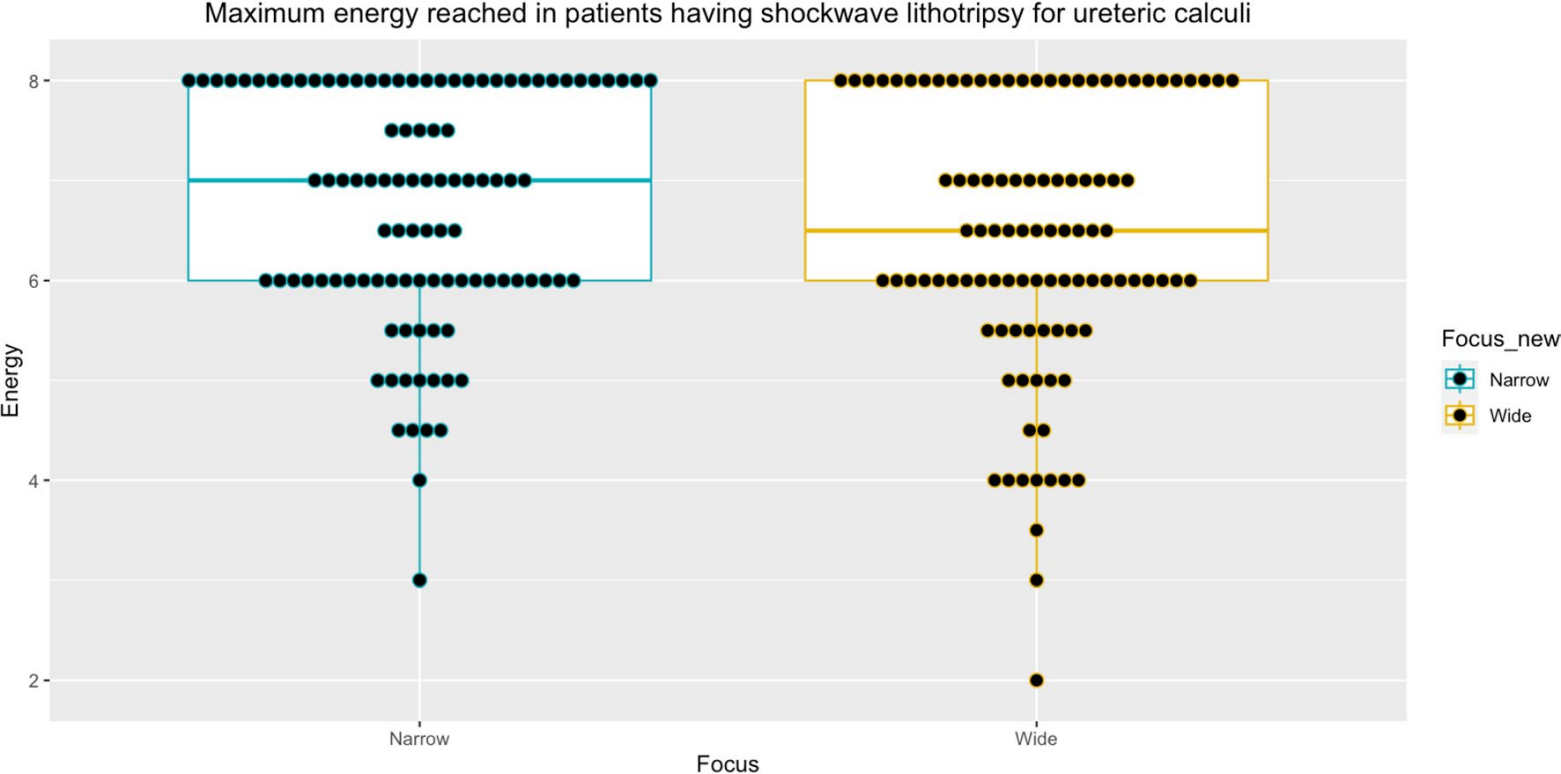
Comparison of maximum energy reached between wide and narrow focus SWL in the ureteric subset groups (*n* = 102 pairs). Median maximum energy reached in ureter subset group = 6.50 (IQR 6.00–8.00), median maximum energy reached in narrow-focus ureter subset group = 7.00 (IQR 6.00–8.00),median maximum energy reached in wide-focus ureter subset group = 6.50 (IQR 6.00–8.00). P narrow vs. wide = 0.18

After matching patients based on the number of SWL treatments received, the size of stone treated, and the site of the stone within the kidney or ureter, 152 paired data sets were identified for analysis (Table 2). Calculi measured between 4-14 mm (median stone size 6 mm, interquartile range (IQR) 5-7 mm). The most common stone location was the distal ureter (56/152 = 36.84% pairs) followed by the proximal ureter (38/152 = 25% pairs) (Table 2). There was an equal distribution of stones in the middle and lower renal calyces (21/152 = 13.82% pairs in each) and in the upper calyx and pelvi-ureteric junction (8/152 = 5.26% pairs each) (Table 2).” Maximum energy delivered was comparable between those treated with narrow- and wide-focus SWL (median maximum energy level = 6 NF-SWL and WF-SWL, *p* = 0.08, Figs. 2, 3 and 4).

**Table 2:** Stone demographics of the paired patient population. Total number of matched pairs was 152

Parameter	Number of pairs
*Number of treatments*	
1	59
2	87
3	3
4	3
*Size of stone (mm)*	
4	13
5	35
6	38
7	33
8	14
9	11
10	6
11	0
12	1
13	0
14	1
*Site*	
Upper calyx	8
Middle calyx	21
Lower calyx	21
Pelvi-ureteric junction	8
Proximal ureter	38
Distal ureter	56

### Stone clearance

Rates of complete stone clearance were higher for patients treated with a wide focus in comparison to those treated with narrow focus when considering all stones (WF-SWL = 84 vs. NF-SWL = 63, *p* = 0.04, Table 3). However, no differences in rates of overall treatment success were identified between wide and narrow-focus groups. Thus, in the overall cohort, 113/152 (74.3%) of the wide-focus group and 100/152 (65.8%) of the narrow-focus group had clinically-acceptable stone fragmentation following SWL (clinically-acceptable stone fragmentation in renal stones group: wide = 34/50 (68.0%), narrow = 27/50 (54.0%) in the renal stones group; clinically-acceptable stone fragmentation in ureteric stones group: wide = 79/100 (79.0%), narrow = 73/100 (73.0%))(Table 3). In the overall cohort, 39/152 (25.7%) of the wide-focus group and 52/152 (34.2%) of the narrow-focus group required further treatment after SWL (requirement for further treatment in renal stones group: wide = 16/50 (32.0%), narrow = 23/50 (46.0%); requirement for further treatment after SWL in ureteric stones group: wide = 23/100 (23.0%), narrow = 29/100 (29.0%))(Table 3).

**Table 3:** Comparison of stone clearance between patients undergoing wide and narrow focus shockwave lithotripsy for renal or ureteric calculi. Treatment success indicates insignificant fragments only remaining and no need to proceed to clinic to discuss alternative treatment options. Statistically significant p values following correction for multiple testing highlighted in bold

	All stones *N* = 152 pairs			Renal stones *n* = 50 pairs			Ureteric stones *n* = 102 pairs		
	Wide	Narrow	*p*	Wide	Narrow	*p*	Wide	Narrow	*p*
Complete stone clearance	84	63	**0.04**	21	12	0.58	63	51	0.41
Treatment success	113	100	0.20	34	27	1.00	79	73	1.00
Persistent stones, alternative treatment offered	39	52	0.20	16	23	0.87	23	29	1.00

### Complications

There were no differences in rates of re-presentation to hospital, SWL-related symptoms, complications, or need for further treatment between patients who underwent SWL with a wide focus vs. narrow focus (Table 4). No patient in either the wide or narrow focus group had a haematoma.

**Table 4:** Comparison of rates of re-presentation to hospital, symptoms, complications, and need for further treatment within 31 days of SWL between patients undergoing wide and narrow focus shockwave lithotripsy for renal and ureteric calculi

		All stones *n* = 152 pairs			Renal stones *n* = 50 pairs			Ureteric stones *n* = 102 pairs		
		Wide	Narrow	*p*	Wide	Narrow	*p*	Wide:	Narrow	*p*
Re-presentation	Unplanned healthcare reattendance	27	19	1.00	12	8	1.00	15	11	1.00
	Readmission	6	5	1.00	2	2	1.00	4	3	1.00
	Admission directly from SWL	4	0	1.00	1	0	-	3	0	-
	Pain	26	17	1.00	11	7	1.00	15	10	1.00
Symptoms	Fever	5	4	1.00	1	2	1.00	4	2	1.00
	Haematuria	8	7	1.00	3	2	1.00	5	5	1.00
	Other	9	4	1.00	3	3	1.00	6	1	1.00
Complications	Urinary tract infection	10	2	0.29	5	2	1.00	5	0	1.00
	Haematoma	0	0	-	0	0	-	0	0	-
	Other	5	2	1.00	1	0	1.00	4	2	1.00
	Need for analgesia	26	12	0.64	10	5	1.00	16	7	1.00
Treatment	Need for antibiotics	11	4	0.46	5	3	1.00	6	1	1.00
	General anaesthetic procedure	2	2	1.00	1	1	1.00	1	1	1.00
	Other	6	6	1.00	1	2	1.00	5	4	1.00

## Discussion

This service evaluation suggests that there is no difference in overall treatment success rates between wide focus-SWL and narrow focus-SWL (treatment success 74% WF-SWL, 66% NF-SWL *p* = 0.20). A recent randomised control trial comparing narrow (2 mm) and wide (8 mm) focus SWL-size reported comparable stone-free rates one month after three sessions of SWL (86.6% NF-SWL vs. 86.8% WF-SWL) [13]. Energy usage per SWL treatment was comparable between wide and narrow focus groups suggesting that any improved stone fragmentation by WF-SWL is attributable to focus size rather than greater energy delivery. Experimental studies and computer modelling have shown that when the size of SWL-focus exceeds that of a stone, shockwaves travel along the outer stone surface, generating shear stress and driving fragmentation, which may account for our findings [5, 14]. Furthermore, a wider field of focus would increase the chances of shockwaves hitting a calculus in situations where there is movement. Respiratory effort is thought to account for 7.7 mm (+/- 2.9 mm) and 3.6 mm (+/- 2.1 mm) movement of renal and ureteric stones, respectively [15] and Sorensen et al. describe that up to 40% of lithotripsy shockwaves miss the targeted stone [16].

Treatment guidelines for renal and ureteric stones include SWL and endoscopic options stratified according to stone size [11, 17, 18]. While retrograde ureteroscopic and percutaneous approaches to renal and ureteric stones are available, rates of major complications in our analysis were lower than those reported in the literature for these methods [19]. No differences in rates of complications up to 31 days post-SWL were detected between wide and narrow focus patient groups. Urinary calculi may contain bacteria within their matrix; various studies have isolated bacteria from 15 to 70% of stones, with the ubiquity and organisms varying by stone composition [20, 21]. For calculi harbouring bacteria within their matrix, destruction could prompt their release into the urine, potentially increasing the risk of urinary tract infection (UTI). Our results suggest that although rates of UTI and antibiotic use were numerically higher with WF-SWL, statistical significance was not reached in this small cohort (UTI; WF-SWL = 10, NF-SWL = 2, *p* = 0.29; antibiotic prescribing; WF-SWL = 11, NF-SWL = 4, *p* = 0.46).

In contrast to previous findings describing greater pain associated with NF-SWL use [13], we identified no differences in requirement for analgesia between the treatment groups (WF-SWL = 26, NF-SWL = 12, *p* = 0.64).

In clinical practice, choice of focus size may be influenced by source-to-target distance, with shorter distances favouring narrow focus fields [22]. Rising levels of adiposity worldwide [23] would increasingly favour the use of wide focus settings. A subgroup analysis comparing efficacy of wide and narrow focus shockwaves in groups of patients categorised by BMI, waist circumference, or skin-to-stone distance could offer more insight.

Our findings are limited by the retrospective nature of analyses and paucity of data on key factors known to affect SWL outcomes, such as stone density, composition, and skin-to-stone distance. Including these factors may provide a more accurate assessment of the impact of focus size on stone clearance. Furthermore, pairs were also not matched for gender, age, or stone laterality [24]. We recognise that the endourological community has not reached consensus regarding the definition of clinically insignificant fragments and that some urology units would not deem the persistence of fragments < 4 mm as “treatment success”. Future studies of adequate power should randomise patients, stratified by stone location, to wide and narrow SWL treatment and prospectively collect data including energy delivery, number of shocks required, number of treatments required, image-defined stone clearance, adverse events, and requirement for requirement for further treatment after SWL to establish the relative efficacy of wide and narrow focus SWL approaches.

## Conclusions

In summary, this retrospective service evaluation demonstrates no differences in rates of overall treatment success nor complications on comparing WF-SWL and NF-SWL. Randomised clinical trials may be of value in further evaluating the relevance of SWL-focus size.

## Data Availability

No datasets were generated or analysed during the current study.
